# Identification of an interaction between calcium-dependent protein kinase 4 (*Et*CDPK4) and serine protease inhibitor (*Et*Serpin) in *Eimeria tenella*

**DOI:** 10.1186/s13071-018-2848-y

**Published:** 2018-04-23

**Authors:** Ling Lv, Bing Huang, Qiping Zhao, Zongping Zhao, Hui Dong, Shunhai Zhu, Ting Chen, Ming Yan, Hongyu Han

**Affiliations:** 1 College of Life and Environment Sciences, Shanghai Normal University, Shanghai, 200234 China; 2 Shanghai Veterinary Research Institute, Chinese Academy of Agricultural Sciences, Key Laboratory of Animal Parasitology of Ministry of Agriculture, Minhang, Shanghai, 200241 People’s Republic of China

**Keywords:** *Eimeria tenella*, Calcium-dependent protein kinases, Serpin, Yeast two-hybrid, Bimolecular fluorescence complementation, Co-immunoprecipitation

## Abstract

**Background:**

*Eimeria tenella* is an obligate intracellular apicomplexan protozoan parasite that has a complex life-cycle. Calcium ions, through various calcium-dependent protein kinases (CDPKs), regulate key events in parasite growth and development, including protein secretion, movement, differentiation, and invasion of and escape from host cells. In this study, we identified proteins that interact with *Et*CDPK4 to lay a foundation for clarifying the role of CDPKs in calcium channels.

**Methods:**

*Eimeria tenella* merozoites were collected to construct a yeast two-hybrid (Y2H) cDNA library. The Y2H system was used to identify proteins that interact with *Et*CDPK4. One of interacting proteins was confirmed using bimolecular fluorescence complementation and co-immunoprecipitation *in vivo*. Co-localization of proteins was performed using immunofluorescence assays.

**Results:**

Eight proteins that interact with *Et*CDPK4 were identified using the Y2H system. One of the proteins, *E. tenella* serine protease inhibitor 1 (*Et*Serpin), was further confirmed.

**Conclusion:**

In this study, we screened for proteins that interact with *Et*CDPK4. An interaction between *Et*Serpin and *Et*CDPK4 was identified that may contribute to the invasion and development of *E. tenella* in host cells.

## Background

*Eimeria tenella* is an obligate intracellular apicomplexan protozoan parasite that causes huge economic losses in the poultry industry. Protozoans have complex life-cycles and need to invade host cells to grow, develop and reproduce. Invasion is a multi-step process that involves the formation, in most cases, of parasitophorous vacuoles within the host cells in which the parasites replicate [1]. Successful invasion of and subsequent escape from host cells, as well as spreading within the host, are important events in the establishment of parasite infections.

Calcium (Ca^2+^) plays an important role in regulating parasite protein secretion, movement, differentiation, invasion and escape from host cells [2]. In eukaryotic cells, Ca^2+^ is an important signaling molecule, acting as a second messenger and regulating many physiological processes in the body [3]. When cells are stimulated by hormones or electricity, cytoplasmic Ca^2+^ concentrations increase, causing a series of intracellular physiological responses [4]. Calcium-dependent protein kinases (CDPKs) are effectors of Ca^2+^ signaling that play important roles in cells. Recently, CDPKs have been found in plants, green algae and apicomplexan protozoans but have not been reported in bacteria, nematodes, fungi or vertebrates [5]. In cells, CDPKs phosphorylate substrate proteins to produce an amplification cascade reaction that transmits the Ca^2+^ signal. CDPKs have four domains: a variable region, a catalytic region, a link region, and a regulatory region. The catalytic zone can bind to ATP and serine or threonine residues of phosphorylated substrates. In the absence of Ca^2+^ ions, the linker region binds to the catalytic zone of the substrate and inhibits kinase activity. The regulatory region is a Ca^2+^ binding zone with EF chiral structure, which allows for CDPKs to be highly compatible with Ca^2+^ and not dependent on calmodulin [6].

In apicomplexan protozoans, CDPKs have been identified as part of the mechanistic link between Ca^2+^ signaling and differentiation, motility, invasion and escape from the host cell [2, 7]. Different CDPKs have specific expression patterns at different developmental stages and they regulate many Ca^2+^-dependent physiological processes. For example, in *Plasmodium falciparum*, *Pf*CDPK1 can phosphorylate Myosin A tail domain-interacting protein (MTIP) and glideosome-associated protein 45 (GAP45), which are the components of the motor complex that providing a driving force for parasites to invade the host [8]. The peptide P3, a part of the *Pf*CDPK1 junction domain, inhibits the activity of CDPK1 and the secretion of microneme proteins during the invasion of erythrocytes by *P. falciparum* merozoites, indicating that CDPK1 is a key regulatory molecule during movement and invasion of host cells by asexual blood stage *P. falciparum* parasites [9]. Similar findings have been reported in *Toxoplasma gondii.* Conditional suppression of *Tg*CDPK1 results in a block of essential phenotypes, including parasite motility and host cell invasion and escape, indicating that *Tg*CDPK1 controls Ca^2+^-dependent secretion of microneme proteins [10]. *Tg*CDPK1 exploits ATP-binding pockets to recognize its substrates, which include the dynamin-related protein DrpB [11]. *Tg*CDPK7 knockdown parasites show significant growth defects and do not progress through cell division. Additionally, *Tg*CDPK7 affects the partitioning and number of centrosomes during parasite division and the polarity of budding, which illustrates that *Tg*CDPK7 is necessary to maintain the distribution and localization of centrosomes in *T. gondii*, an essential process for survival during the breeding stage [12].

Recent studies on *E. tenella* CDPK members have suggested that CDPKs regulate biological functions in *E. tenella* [13–15]. In a preliminary study of its function, it was found that *Et*CDPK3 was localized to the apical end of sporozoites during the initial invasion stage. Specific antibodies blocking *Et*CDPK3 inhibit host invasion, indicating that *Et*CDPK3 participates in host cell invasion and development within the host [16]. In our previous report, another *E. tenella* CDPK, *Et*CDPK4, was found to be highly expressed during the merozoite stage, although transcriptome levels of *Et*CDPK4 were highest during the sporozoite stage. Inhibiting the activity of *Et*CDPK4 reduced sporozoite invasion, indicating that *Et*CDPK4 participates in host cell invasion [15]. However, the *Et*CDPK4 regulatory mechanisms and targets in *E. tenella* remain unclear.

Analyses of protein-protein interactions (PPIs) are crucial for the study of various cellular processes and protein function [17]. To further understand the function of *Et*CDPK4, we conducted yeast two-hybrid (Y2H) screening and identified an interaction between *Et*CDPK4 and *E. tenella* serine protease inhibitor 1 (*Et*Serpin). Moreover, we confirmed the interaction between *Et*CDPK4 and *Et*Serpin in DF-1 cells by co-immunoprecipitation (Co-IP) and bimolecular fluorescence complementation (BiFC).

## Methods

### Antisera and recombinant plasmids

The following antibodies used for immunoblotting and immunofluorescence assays (IFAs) were prepared and stored at -20 °C in the laboratory: anti-*Et*CDPK4 rabbit and mouse antisera [15], anti-*Et*Serpin rabbit antisera are described elsewhere [18]. The recombinant plasmid pCAGGS-*Et*Serpin was a gift from Dr. Ye Wang, stored at the laboratory.

The sequences of primers used for PCR are provided in Table 1. To express fusion proteins, *Et*CDPK4 (ETH_00010685) was cloned into pcDNA3.1-flag (Biovector, Cambridge, MA, USA) and pBIFC-VC155 vectors and the recombinant plasmids were designated pCDNA3.1-flag-*Et*CDPK4 and pBIFC-VC155-*Et*CDPK4. *EtSerpin* was cloned into the *Sal*I and *Xho*I sites of pBIFC-VN155 using a ClonExpress kit (Vazyme, Nanjing, China).

**Table 1:** Sequences of primers used in this study

Primer ID	Primer sequence (5'-3')
pBiFC-VN155-Serpin UP	ATGGCCATGGAGGCCCGAATTCGGGCCACCATGGCTCTGTTGAGTAAGCT
pBiFC-VN155-Serpin LOW	ACGCCGGACGGGTACCTCGAGCTGCTGTGCTGCTGCCGG
pBiFC-VC155-CDPK4 UP	GCGAATTCGGGCCACCATGGAGCAGGTGATGGGTGGGCGGGAGGT
pBiFC-VC155-CDPK4 LOW	GCGGTACCAAATTCGTCCCAGTCAATCTGCCCAT
pCDNA3.1-flag-CDPK4 UP	CGCGGATCCATGGAGCAGGTGATGGGTGGGCGGGAGGT
pcDNA3.1-flag-CDPK4 LOW	GCGAATTCCTAAAATTCGTCCCAGTCAATCTGCCCAT

### Parasite

*Eimeria tenella* (CAAS211116-11) was obtained from the Shanghai Veterinary Research Institute, Chinese Academy of Agricultural Sciences. The parasite was obtained as previously described [19] by inoculation 2-week-old chickens which were free of infection before experimental inoculation.

Unsporulated oocysts were obtained from the cecal contents of chickens infected with 1 × 10^4^ *E. tenella* sporulated oocysts at 8 days post-infection (p.i.). Sporulated oocysts were harvested from the unsporulated oocysts which underwent sporulation with 2.5% potassium dichromate at 28 °C for 72–120 h in the presence of oxygen. Sporozoites were excysted with trypsin and chicken bile *in vitro* and purified from cleaned, sporulated oocysts by chromatography over columns of nylon wool and DE-52 cellulose as previously described [19]. Second-generation merozoites (merozoites II) were isolated from the cecum and the cecal contents of chickens 115 h after infection *E. tenella* and then purified with Percoll [20].

### Construction of a Y2H cDNA library of *E. tenella* merozoites II

Yeast strains Y187 and Y2H Gold (Takara, Tokyo, Japan) used for the Y2H screen were prepared according to Yeastmaker^TM^ Yeast Transformation System 2 User Manual (Clontech, Mountain View, CA, USA).

Total RNA was isolated from merozoites II of *E. tenella* with Trizol (Life Technologies, Carlsbad, CA, USA) according to the manufacturer’s instructions. PolyA^+^ was purified with a PolyA^+^ Tract mRNA Isolation System kit (Promega, Madison, WI, USA) after the quality of total RNA was assessed. The Y2H cDNA library of *E. tenella* merozoites was constructed using a Make Your Own “Mate & Plate” Library System kit (Clontech, Mountain View, CA, USA). First-strand cDNA was synthesized and then amplified into double-stranded cDNAs (dscDNAs) using long distance PCR. dscDNAs shorter than 200 bp were removed using a Chroma Spin column (Takara, Tokyo, Japan). dscDNAs were cloned into the pGADT7-Rec vector (Clontech, Mountain View, CA, USA). The resulting plasmids were transformed into the Y187 yeast strain according to the instructions. To determine the transformation efficiency, 100 μl of 1:10,000, 1:1000, 1:100 and 1:10 dilutions were spread on 100-mm plates containing synthetic dropout (SD)/-Leu media and incubated at 30 °C for 3−5 days. The remainder was spread on another SD/-Leu plate and the library solution was collected. To calculate the library size, 100 μl of 1:10,000, 1:1000, 1:100 and 1:10 dilutions of the library solution were spread onto media and incubated at 30 °C for 3−5 days. Fifty-one colonies were randomly selected for PCR identification and analysis of library insert size and recombination efficiency.

### Y2H library screening

*Et*CDPK4 cDNA was inserted in-frame with the GAL4 DNA-binding domain into the vector pGBKT7-GAL4 to construct the recombinant plasmid pGBKT7-*Et*CDPK4. A non-autoactivating and non-toxic pGBKT7-*Et*CDPK4 were acquired and bait proteins were expressed [21]. For interaction mating, the bait protein and the library were co-cultured at 30 °C with shaking for 20 h then spreading onto SD/-Leu/-Trp/-His/-Ade (QDO) selection media. After mating, clones were transferred to QDO media. Blue clones were confirmed by culturing on SD/-Leu/-Trp/-His/-Ade supplemented with X-α-gal (QDO/X) media and then cultured on SD/-Leu/-Trp/-His/-Ade supplemented with X-α-gal and aureobasidin A (QDO/X/A) media. Only those clones growing on QDO/X/A media were further characterized. Confirmation of interacting clones was performed by sequencing and non-target plasmids were eliminated. Positive clones were further confirmed by prey plasmid rescue and re-transformation into Y2H Gold with pBDGAL4-*Et*CDPK4 or with the negative control plasmid (empty pBD-GAL4).

### Immunolocalization

Purified, freshly excysted sporozoites and merozoites II were incubated in phosphate-buffered saline (PBS), transferred to a glass slide, and air-dried as previously described [22]. The chicken embryo fibroblast (DF-1) cell line was cultured in Dulbecco’s Modified Eagle’s Medium (DMEM) (Gibco, Grand Island, NY, USA) supplemented with 10% fetal bovine serum (FBS) and 1% penicillin/streptomycin. 2.0 × 10^5^ cells per well with slices were used for parasite invasion [18]. Purified sporozoites were washed three times with sterile PBS, infected into DF-1 cells then cultured at 41 °C for 2 h. The cells cultured with slices were collected and washed with PBS. The slices were air-dried and fixed in 4% paraformaldehyde for 20 min, then permeabilized with 0.1% Triton X-100 in PBS for 20 min and incubated with 2% bovine serum albumin (BSA) in PBS overnight at 4 °C. At dilutions of *Et*CDPK4 and *Et*Serpin antisera for 1 h, a 1:500 dilution of goat anti-rabbit IgG fluorescein isothiocyanate (FITC)-conjugated antibody (Sigma, St. Louis, MO, USA) and goat anti-mouse IgG cyanine (Cy3)-conjugated antibody (Sigma, St. Louis, MO, USA) were added and incubated for 1 h at 37 °C. 4,6-diamidino-2-phenylindole (10 μg/ml, Beyotime, Haimen, China) was used to stain nuclei for 30 min. After each step, slides were washed three times with PBS. 50 μl Fluoromount Aqueous Mounting Medium (Sigma, St. Louis, MO, USA) was added before observation under a fluorescence microscope (Olympus, Tokyo, Japan).

### BiFC assay

For BiFC assays, cells must take up the expression vector. Therefore, IFAs were performed to confirm expression of the fusion proteins in the DF-1 cells. The recombinant plasmids VC155-*Et*CDPK4 and VN155-*Et*Serpin were transfected into 6.0 × 10^5^ DF-1 cells and cultured in six-well plates for 24 h. Briefly, 4 μg plasmid DNA and 10 μl lipofectamine 2000 (Invitrogen, Carlsbad, CA, USA) were mixed, incubated at room temperature for 20 min, and gently added to the cells. After 6 h, the DNA transfection reagent was replaced with DMEM containing 2% FBS and 200 U/ml penicillin/streptomycin. For IFAs, DF-1 cells transfected with recombinant plasmids were fixed in 2% paraformaldehyde for 20 min. The samples were permeabilized with 0.1% Triton X-100 in PBS pH7.4 for 20 min and then blocked with 2% BSA in PBS at 4 °C overnight. The relevant antisera and the goat anti-rabbit secondary antibodies were used for incubation. Finally, the samples were visualized using a fluorescence microscope.

After confirming that the DF-1 cells could take up the two constructs, the recombinant plasmids VC155-*Et*CDPK4 and VN155-*Et*Serpin were co-transfected into DF-1 cells. 30 h later, the cells were observed under an inverted fluorescence microscope. Two non-fluorescent fragments, pBiFC-bfosVC155 and pBiFC-bjunVN155, form a fluorescent complex that can be detected using a fluorescence microscope were used as positive controls. Two non-fluorescent fragments, pBiFC-bfosVC155 (delta ZIP) and pBiFC-bjunVN155, were used as negative controls.

### Co-IP

To confirm their expression in DF-1 cells, the recombinant plasmids pCDNA3.1-flag-*Et*CDPK4 and pCAGGS-*Et*Serpin were transfected into 2.0 × 10^6^ DF-1 cells and analyzed by western blot. After confirming that the DF-1 cells could take up the two constructs, the recombinant plasmids were co-transfected into cells as described above. pCDNA3.1-flag and flag-*Et*CDPK4 were used as controls. After 48 h, the transfected cells were washed twice with PBS and lysed with RAPI buffer, cell debris was removed by centrifugation at 12,000× *rpm* for 10 min. The Co-IP assay was performed using the Pierce Co-Immunoprecipitation kit (Thermo, Waltham, MA, USA) following the manufacturer’s instructions using antisera to *Et*CDPK4 for coupling to the resin. Samples were analysed by SDS-PAGE followed by Western blotting then detection with *Et*Serpin antisera or anti-flag antibody. The control was incubated with anti-Flag antibody only. The nitrocellulose membranes were incubated with anti-mouse fluorescent secondary antibody for anti-Flag and anti-rabbit fluorescent secondary antibody for *Et*Serpin antibodies.

## Results

### *Eimeria tenella* merozoite II Y2H cDNA library construction

**Fig. 1:**
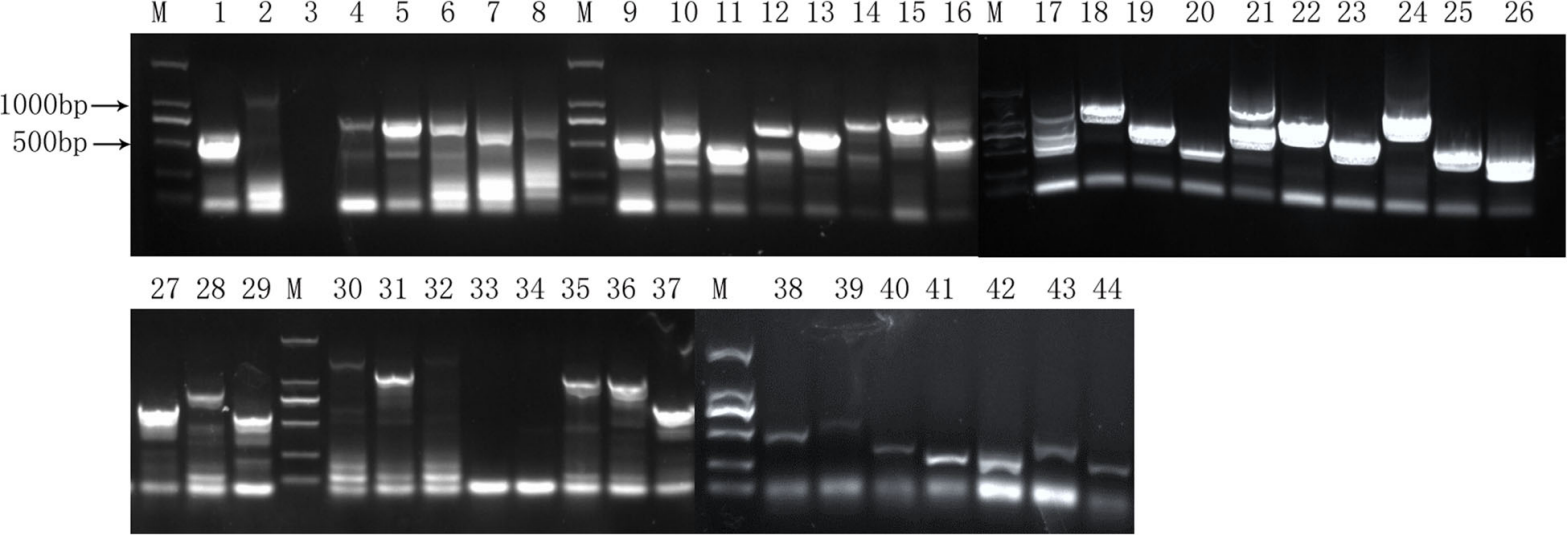
Partial PCR products of randomly selected colonies from the Y2H cDNA library analyzed with electrophoresis on a 1% agarose gel. Lanes 1−44: individual recombinant colonies; Lane M: DNA size marker

Merozoite II cDNA from *E. tenella* was cloned into the pGADT7-rec vector. The resulting plasmids were transformed into the Y187 strain and spread onto SD/-Leu plates. Random clones were selected for PCR analysis (Fig. 1). Results showed that 93.2% of recombinants carried DNA sequences with an average length of 500 bp from *E. tenella* merozoites. The size of the library was 9.6 × 10^9^ CFU and the transformation efficiency of the library was 4.1 × 10^5^ cfu/μg pGADT7-Rec, which was sufficient for subsequent Y2H screening.

### Y2H screening for proteins interacting with *Et*CDPK4

**Fig. 2:**
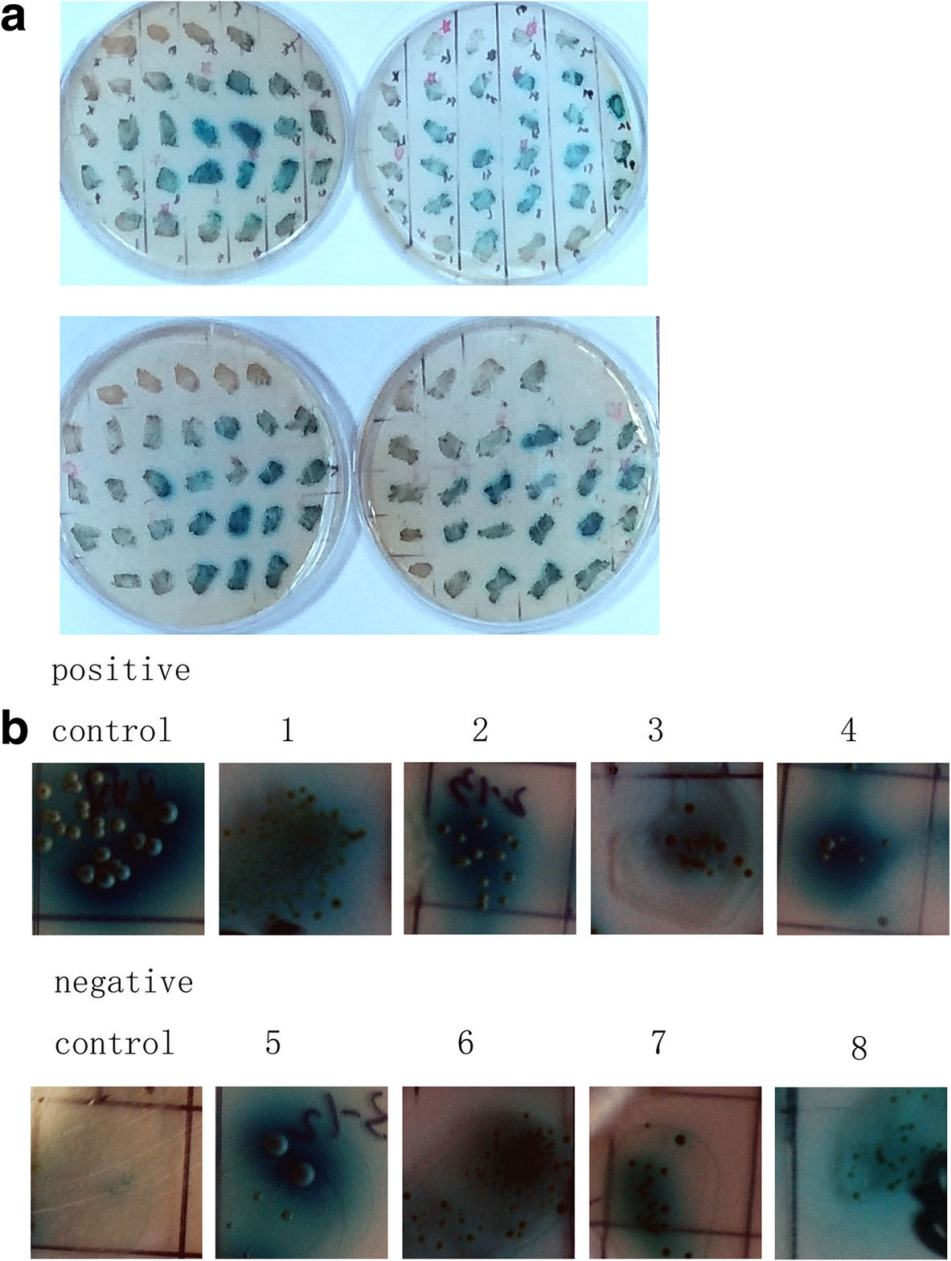
Y2H screen for proteins that interact with *Et*CDPK4. **a** Blue clones acquired by culturing on QDO/X/A media. **b** Positive plasmids confirmed by transforming Y2HGold with pGADT7-*Et*CDPK4

Y2H screening of the *E. tenella* merozoite II cDNA library with *Et*CDPK4 as bait resulted in 69 blue colonies formed (Fig. 2a). Comparison of the DNA sequence of the positive plasmids with the genome of *E. tenell*a (http://www.genedb.org) showed that 30 different *E. tenella* proteins were represented. To test whether these proteins interacted with *Et*CDPK4 in yeast, the positive plasmids were transformed into Y2H Gold using pGADT7-*Et*CDPK4 and spread onto QDO/X/A plates. In total, eight blue colonies (Table 2) formed that contained proteins that interacted with *Et*CDPK4 in the Y2H system (Fig. 2b).

**Table 2:** *Et*CDPK4 targets identified by Y2H screening

Gene ID	Annotation	MW (kDa)
ETH_00011330	SERPIN1 protein	45.5
ETH_00024500	Hypothetical protein	92.3
ETH_00018145	Hypothetical protein	17.9
ETH_00002065	Hypothetical protein	38.4
ETH_00009380	DNA-directed RNA polymerases I and III subunit RPAC1	36.4
EMH_00033980	Hypothetical protein	44.1
ETH_00021190	Sec63 domain-containing DEAD/DEAH box helicase	246.7
ETH_00007745	apical membrane antigen-1	58.0

### Co-localization of *Et*CDPK4 and *Et*Serpin

**Fig. 3:**
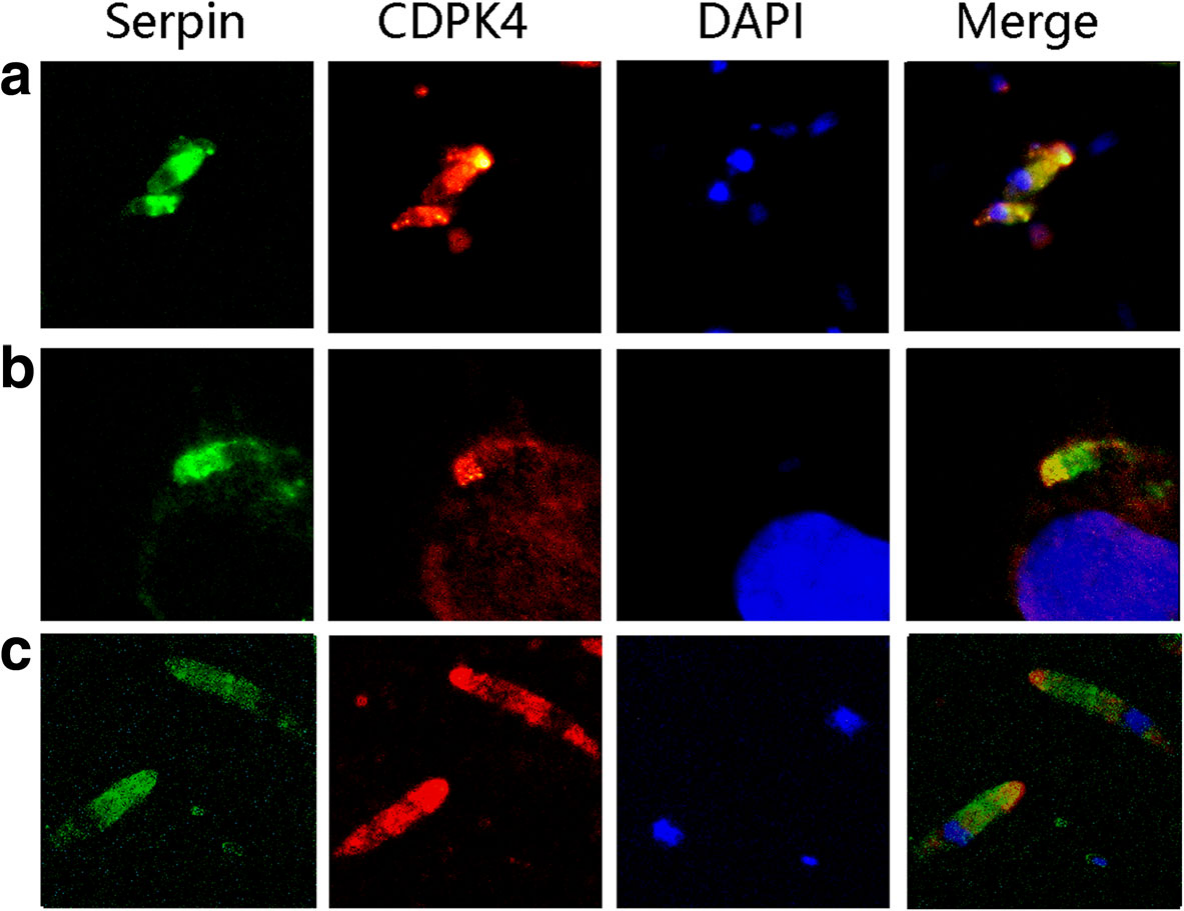
Co-localization of *Et*CDPK4 and *Et*Serpin. **a** IFA performed using antisera against *Et*CDPK4 and *Et*Serpin in sporozoites. **b** IFA performed using antisera against *Et*CDPK4 and *Et*Serpin at 2h PI. **c** IFA performed using antisera against *Et*CDPK4 and *Et*Serpin in merozoites II . *Et*Serpin antibody reactivity was detected with FITC and anti-*Et*CDPK4 reactivity with Cy3 conjugated secondary antibodies

IFAs were performed to determine the location of *Et*CDPK4 and *Et*Serpin. Sporozoites and merozoites II treated with anti-*Et*CDPK4 mouse antisera and anti-*Et*Serpin rabbit antisera showed *Et*CDPK4 and *Et*Serpin distributed throughout the cytoplasm (Fig. 3a, c). At 2 h p.i. of DF-1 cells by sporozoites both proteins of *Et*CDPK4 and *Et*Serpin were at the apical end (Fig. 3b). The co-localization indicated that the proteins of *Et*CDPK4 and *Et*Serpin function in the same location.

### Interaction between *Et*CDPK4 and *Et*Serpin assessed by BiFC

**Fig. 4:**
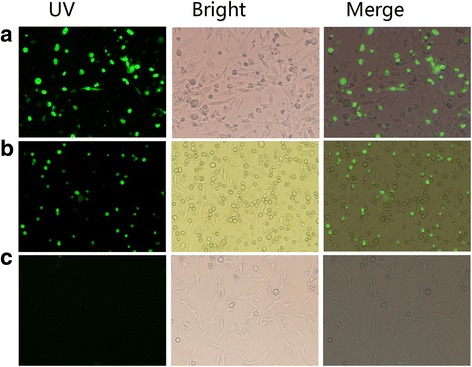
Interaction between *Et*CDPK4 and *Et*Serpin in DF-1 cells assessed by BiFC. VC155-*Et*CDPK4 and VN155-*Et*Serpin were co-transfected into DF-1 cells. **a** Positive controls bFos and bJun co-transfected into DF-1 cells. **b** *Et*CDPK4 and *Et*Serpin co-transfected into DF-1 cells. **c** Negative controls bFos (delta ZIP) and bJun co-transfected into DF-1 cells

Expression of the plasmids VC155-*Et*CDPK4 and VN155-*Et*Serpin in DF-1 cells was confirmed using IFA (data not shown). The recombinant plasmids VC155-*Et*CDPK4 and VN155-*Et*Serpin were then co-transfected into DF-1 cells and observed with an inverted fluorescence microscope. When the positive controls bFos and bJun were transiently co-expressed in DF-1 cells, a positive BiFC signal was detected in the cells. A similar positive BiFC signal was detected when VC155-*Et*CDPK4 and VN155-*Et*Serpin were transiently co-expressed in DF-1 cells, indicating that *Et*CDPK4 and *Et*Serpin interact in DF-1 cells (Fig. 4). In contrast, the negative controls bFos (delta ZIP) and bJun did not produce a detectable fluorescent signal.

### Interaction between *Et*CDPK4 and *Et*Serpin assessed by Co-IP

**Fig. 5:**
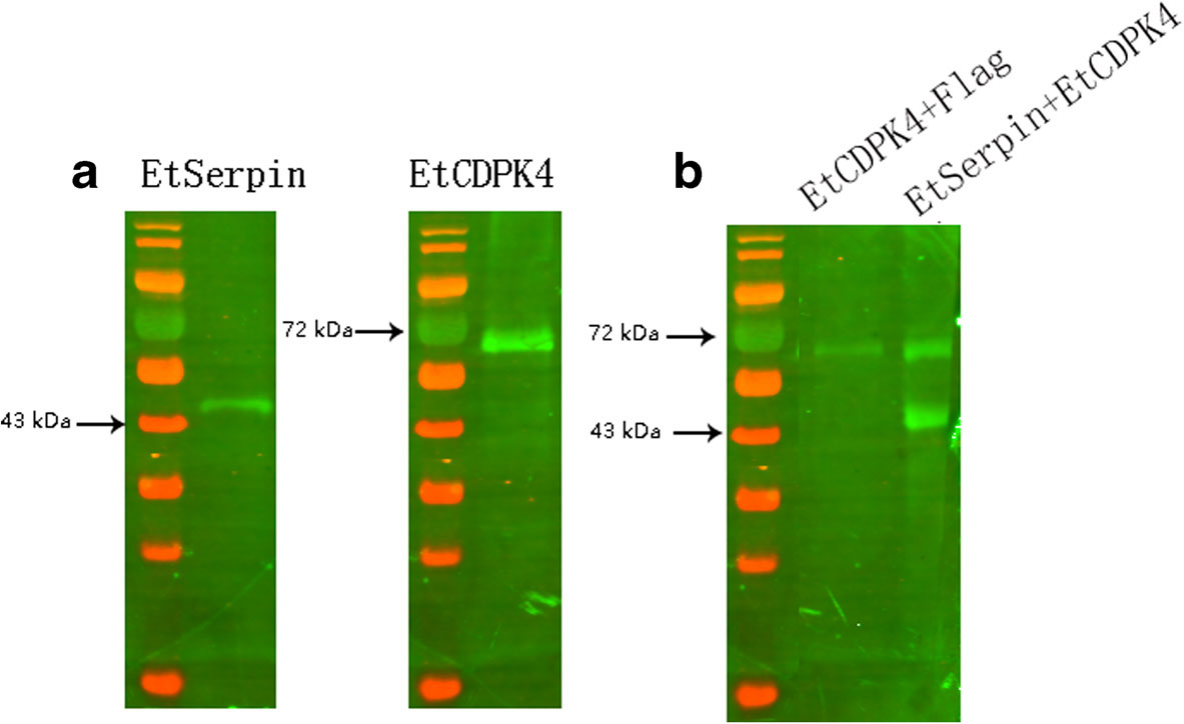
Interaction between *Et*CDPK4 and *Et*Serpin assessed with Co-IP. **a** DF-1 cells were transfected with pCDNA3.1-flag-*Et*CDPK4 and pCAGGS-*Et*Serpin and cellular lysates were analyzed with immunoblotting with antisera against *Et*CDPK4 and *Et*Serpin. **b** The Co-IP was performed using immobilised antisera against *Et*CDPK4. Detection of eluted proteins on immunoblots was by *Et*Serpin and/or anti-flag for *Et*CDPK4 antibodies

Western-blotting results showed that the fusion protein flag-*Et*CDPK4 and *Et*Serpin were successfully expressed in DF-1 cells (Fig. 5a). Co-IP assays showed that when flag-*Et*CDPK4 and *Et*Serpin proteins were incubated with resin that was covalently coupled with *Et*CDPK4 antisera, *Et*Serpin was eluted with *Et*CDPK4 from the resin by elution buffer (Fig. 5b). In contrast, when pCDNA3.1-flag was used instead of *Et*Serpin in control experiments, *Et*CDPK4 alone was detected in the eluate (Fig. 5b). Based on these data, we conclude that an interaction exists between *Et*CDPK4 and *Et*Serpin.

## Discussion

Biochemical analysis of protein complexes and identification of their components is fundamental to the understanding of protein function [23, 24]. Currently, several methods for identifying protein interactions exist, including Y2H techniques, Co-IP, BiFC, phage display technology and pull down experiments.

In the present study, we used the Y2H technique to screen for proteins that interact with *Et*CDPK4. High quality libraries are one of the key elements of Y2H screens. In this study, a high quality Y2H cDNA library was constructed using *E. tenella* merozoites II We achieved a recombination rate of 93.2% and a library size of 9.6 × 10^−9^ CFU, which was sufficient for subsequent Y2H screening. A total of eight interacting proteins were identified (Table 2), one of which was previously reported and described as Serpin1 (ETH_00011330) in our lab [18]. Only eight positive interactions were confirmed on a second round screening. There are maybe several reasons: (i) the incorrect folding and/or instability of an AD fusion protein that could interact with its interacting partner, there are maybe some of these AD fusion proteins in the *E. tenella* merozoites II Y2H cDNA library; (ii) the toxicity of some fusion proteins that could affect the viability of transformed cells [25]; (iii) the quality of the library is a key parameter for the success of a screening, although the quality of *E. tenella* merozoites II Y2H cDNA library that we constructed is good, it cannot include all the cDNAs. Some mRNAs encoding putative interacting proteins are expressed at relatively low levels; these proteins might not be identified. In addition there were several identified plasmids (7/30) failed to rescue so there may well be other proteins which interacted with *Et*CDPK4. In future study, we will screen the putative interacting proteins using other methods. The Y2H system may have technical or biological false positives like any assay system [26]. Therefore, we used other methods, including Co-IP, BiFC and co-localization, to further verify the interaction between *Et*CDPK4 and *Et*Serpin.

Detection of PPIs in living cells is particularly important for understanding biological process [27, 28]. One of effective ways for studying PPIs is BiFC [29]. This assay offers several advantages over other techniques such as Y2H. The method enables real-time observation of PPIs in their natural environment, such as in live cells or animals [30, 31]. In addition, the subcellular localization of the PPI can be observed directly from BiFC [31]. So in this study, we used the BiFC to verify the interaction between *Et*CDPK4 and *Et*Serpin.

CDPKs are present in plants, algae, ciliates and apicomplexan parasites. In plants, CDPKs regulate plant development and biotic and abiotic stress responses. The N-terminal domain of CDPK plays a key role in subcellular localization and function [32]. Most CDPKs have myristoylation sites and cysteine residues that allow for N-terminal palmitoylation and contribute to the localization of CDPKs. *Arabidopsis thaliana At*CPK16 is predicted to be localized to the chloroplast based on multiple prediction methods, whereas N-terminal acylation at N-myristoylation and palmitoylation sites prevents localization to the chloroplast [33]. The N-terminus not only determines subcellular localization, but also interacts with target proteins. For example, *Nicotiana tabacum Nt*CDPK1 could phosphorylate the basic leucine zipper transcription factor RSG (repression of shoot growth) in tobacco. A chimeric CDPK containing *Nt*CDPK1 N-terminus fused to *At*CPK9 can also phosphorylate and interact with RSG, although native *At*CPK9 can neither bind nor phosphorylate RSG [34]. Many *Arabidopsis* CDPKs are membrane localized or membrane associated, which is mediated by N-terminal acylation [35]. In *T. gondii*, the substrate of *Tg*CDPK1, DrpB, interacts with CDPK1 at the N-terminal ATP-binding pocket [11]. In *P. falciparum*, *Pf*CDPK7 interacts with phosphatidylinositol 4,5-bisphosphate *via* its pleckstrin homology domain, guiding its subcellular localization [36]. Functional structure prediction indicates that *Et*CDPK4 contains three N-myristoylation sites, an ATP binding domain, and a serine/threonine protein kinase activation site [15]. N-myristoylation sites and ATP binding domain contribute to the subcellular localization and functions of *Et*CDPK4, which include Ca^2+^ signaling and interacting with substrate proteins. We hypothesise that *Et*CDPK4 and *Et*Serpin interact through the ATP binding domain and play a role in sporozoite invasion. The N-terminal myristolation site of *Et*CDPK4 may help the complex of *Et*CDPK4 and *Et*Serpin to locate to the apex near the membrane surface of parasites when sporozoites invade host cells.

*Et*Serpin was one of the putative interacting proteins of *Et*CDPK4. Serine protease inhibitors (serpins) are a class of proteins composed of 300−500 amino acids with a molecular weight between 40–60 kDa. Although intracellular serpins have been reported, most serpins are present in the extracellular environment [37–39]. Over 500 members of the serpin superfamily have been identified in animals, plants, bacteria, archaea and viruses [40]. In mammals, serpins play crucial roles in processes such as blood coagulation and fibrinolysis [38, 41]. Most serpins consist of three β-folds and 8−9 α-helices. The typical serpin structure includes an exposed reactive center loop conformation above the body of the molecule [42]. Most serpins undergo a significant conformational change from the stressed to the relaxed state that can result in inhibition of target proteases. The activity of some small protease inhibitors can be regulated by specific factors. For example, SERPINC1 is a rare inhibitor that inhibits factor Xa with the cofactor heparin in human [43]. SERPINC1, protease and heparin form a stable ternary complex. Therefore, synergistic interactions between serpins and other molecules can result in different roles for serpin proteins. In the case of protein Z dependent protease inhibitors, protein Z as a vitamin K-dependent co-factors to promote the inhibitory activity of the serpin with FXa on negatively charged phospholipid vesicles and calcium [44]. Vaspin is visceral adipose tissue-derived serine protease inhibitor, promoted the phosphorylation of Akt through PI3K signaling pathway [45]. In parasites, Serpin plays an important role in the inflammatory response, regulating host immunity, development and anticoagulation. For example, *Ixodes ricinus* salivary serpin has anticoagulant activity, including coagulation and fibrinolysis inhibition and binds to cells/macrophages and inhibits TNF secretion [46]. In *T. gondii*, SERPIN B3 and B4 act *via* STAT6 activation to inhibit casapase 3, PARP activation, and DNA fragmentation [47]. In this study, Y2H, Co-IP, and BiFC were used to identify an interaction between *Et*CDPK4 and *Et*Serpin. We expect that, *Et*CDPK4 may interact with *Et*Serpin as a cofactor, similar to SERPINC1 or protein Z, the interaction between *Et*Serpin and *Et*CDPK4 may enhance protease inhibitory activity of *Et*Serpin during sporozoites invasion into host cells. At the same time, the phosphorylation of *Et*CDPK4 may be elevated by *Et*Serpin.

In this study, *Et*CDPK4 and *Et*Serpin were located mainly in the cytoplasm of sporozoites and merozoites II. Co-localization experiments showed that *Et*CDPK4 and *Et*Serpin shared the same apical location during the early invasion of sporozoites into DF-1 cells. These results are consistent with previous reports [15, 18]. Another *Et*Serpin has been reported to be detected 24 h p.i. in DF-1 cells *in vitro* [18]. Additionally, *Et*CDPK4 has been detected in the vacuole 12 h p.i. [15]. *In vitro*, sporozoite invasion inhibition assays indicated that polyclonal antibodies against these two proteins can also reduce the ability of *E. tenella* sporozoites to invade host cells [15, 18]. Therefore, we speculate that the interaction between *Et*CDPK4 and *Et*Serpin is likely to play an important role in sporozoite invasion. When the sporozoites invade the host cells, the complex could release into the host cells to inhibit host protease activity which may delay host cell apoptosis.

Although we confirmed the interaction between *Et*Serpin and *Et*CDPK4 with several methods, we also identified but did not confirm other proteins that interact with *Et*CDPK4 using the Y2H screen. The interaction between *Et*Serpin and *Et*CDPK4 may contribute to the invasion of *E. tenella* in host cells, the complex could inhibit host protease activity to delay host cell apoptosis during sporozoite development in host cells. However, further research on the function of the interaction between *Et*Serpin and *Et*CDPK4 at the time of invasion is needed.

## Conclusions

In this study, we constructed a Y2H cDNA library to screen for proteins that interact with *Et*CDPK4. *Et*Serpin was demonstrated to co-localize and interact with *Et*CDPK4, which may promote to the invasion and development of *E. tenella* in host cells.

## Abbreviations

*At*CPK9: *Arabidopsis thaliana* CPK9
BiFC: Bimolecular fluorescence complementation
BSA: Bovine serum albumin
CDPKs: Calcium dependent protein kinase
Co-IP: Co-immunoprecipitation
DMEM: Dulbecco’s Modified Eagle’s Medium
dscDNAs: Double-stranded cDNAs
*Et*CDPK3: *Eimeria tenella* CDPK3
*Et*CDPK4: *Eimeria tenella* CDPK4
*Et*Serpin: *Eimeria tenella* Serpin
FBS: Fetal bovine serum
GAP45: Glideosome-associated protein 45
IFAs: Immunofluorescence assays
MTIP: Myosin A tail domain-interacting protein
*Nt*CDPK1: *Nicotiana tabacum* CDPK1
PBS: Phosphate-buffered saline
*Pf*CDPK1: *Plasmodium falciparum* CDPK1
p.i.: Post-infection
PPI: Protein-protein interactions
QDO: SD/-Leu/-Trp/-His/-Ade
QDO/X: SD/-Leu/-Trp/-His/-Ade supplemented with X-α-gal
QDO/X/A: SD/-Leu/-Trp/-His/-Ade supplemented with X-α-gal and aureobasidin A
*Tg*CDPK1: *Toxoplasma gondii.* CDPK1
YPDA: Yeast peptone dextrose adenine
Y2H: Yeast two-hybrid
